# The hidden costs of using media to mimic the hosts of *Fusarium graminearum*: An epigenetic perspectives

**DOI:** 10.1371/journal.ppat.1014345

**Published:** 2026-07-07

**Authors:** Jade F. Smith, Hans-Wilhelm Nuetzmann, Martin Darino, Kim E. Hammond-Kosack

**Affiliations:** 1 Translating Biotic Interactions, Rothamsted Research, Harpenden, Herts, United Kingdom; 2 Department of Health and Life Sciences, The University of Exeter, Exeter, United Kingdom; University of Maryland, Baltimore, UNITED STATES OF AMERICA

## Abstract

Pathogens dynamically reprogrammed gene expression when transitioning between nonhost and host environments. Epigenetic regulation can provide a rapid and reversible mechanism for this shift. Using published data from Shao et al. (2024) and Zhao et al. (2024), we compare chromatin states in the fungus *Fusarium graminearum* under *in vitro* trichothecene mycotoxin (deoxynivalenol) inducing conditions and during wheat spike infection. This revealed striking differences in H3K4me3 and H3K27me3 landscapes with the two datasets showing limited overlap in marked genes and distinct genomic distributions. This indicates that chemically induced cultures only partially replicate the complex signals encountered *in planta* and emphasise the need for infection-reflective experimental designs to accurately characterise pathogenicity mechanisms.

## Introduction

Pathogens shift between nonhost and host environments, adjusting gene expression to evade detection and enable infection. While transcription factor control is well studied [1,2], recent work shows chromatin structure and epigenetic modifications fine-tune this process [3]. To study these dynamics, pathogens are often grown under infection-mimicking *in vitro* conditions, which are simpler and less costly than host-based experiments [4]. Examples include dental biofilms in artificial saliva [5], human pathogens in tissue culture [6], and plant pathogens in host-like media. For instance, *Fusarium graminearum* (*Fg*), the causal agent of Fusarium head blight (FHB) disease, can be cultured in wheat defence compound-containing media [7]. This report uses *Fg* as a case study to show how epigenetic states shift with environmental cues, comparing *in vitro* growth to *in planta* associated growth. FHB causes global wheat losses of 28 million tonnes annually [8], leading to spike bleaching, shrivelled grains, and contamination with mycotoxins such as the b-type trichothecene, deoxynivalenol (DON), which harms humans and livestock [9,10]. Therefore, understanding these mechanisms of gene expression control is vital for effective control strategies.

Epigenetic regulation enables rapid, targeted shifts in gene expression [11]. This report focuses on histone modifications: chemical changes to histone proteins that shape chromatin structure and transcription [11]. Common marks include methylation and acetylation, which alter DNA accessibility [12,13]. In *Fg*, methylation can activate genes (e.g., H3K4me3, where ‘me3’ denotes trimethylation of lysine 4 on histone H3) or repress them (e.g., H3K27me3, H3K9me3) [12]. Acetylation (e.g., H3K27ac) neutralises lysine charge, promoting open chromatin and transcription in *Fg* [14]. However, the effect on gene expression varies between organisms. Histone methylation and acetylation are added by methyltransferases and acetyltransferases, respectively, and removed by demethylases and deacetylases [11]. These reversible marks create a dynamic chromatin landscape responsive to environmental cues [11]. For example, plant defence signals trigger H3K27me3 loss and gain of activating H3K27ac, causing increased effector expression in the rice blast fungal pathogen *Magnaporthe oryzae* [15].

**Fig 1:**
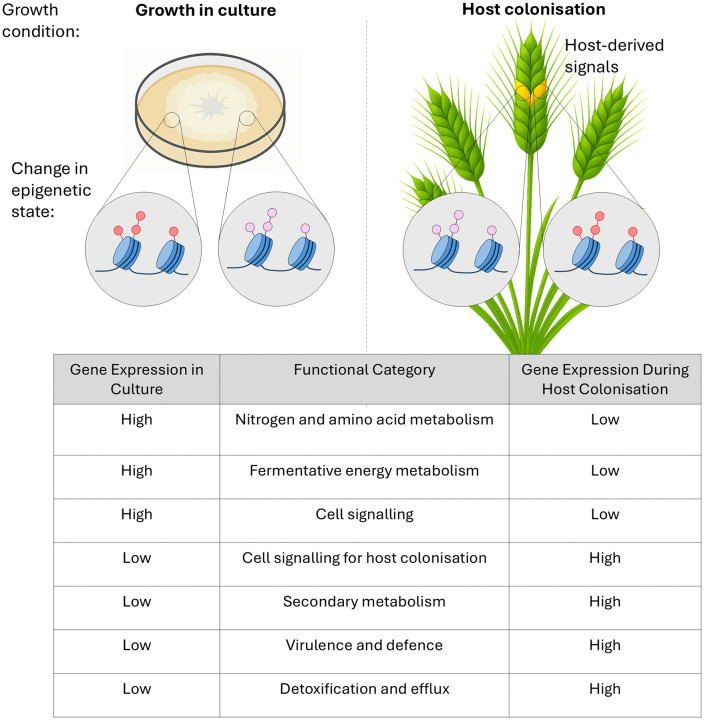
Conceptual diagram illustrating how distinct transcriptional programmes and associated epigenetic states enable *Fusarium graminearum* to adapt to *in vitro* growth versus plant infection and colonisation [17]. Pink circles represent activating epigenetic modifications while red circles represent repressive epigenetic modifications. The specific genes and Functional Category examples included in this Figure have been taken from the comparative study published by Boedi *et al.* [16], where “High” and “Low” refer to gene expression levels.

In culture, *Fg* mainly expresses housekeeping genes for metabolism and growth [16]. Upon host contact, *Fg* rapidly activates genes for colonisation, stress adaptation, and immune evasion [16]. This shift reflects distinct epigenetic landscapes enabling condition-specific transcription (Fig 1). Similar variation occurs across its life cycle, as *Fg* persists on crop residues, as airborne spores, during different infection phases or on alternative hosts with each requiring tailored transcriptional and epigenetic profiles [16]. Furthermore, biosynthetic gene clusters (BGCs) in *Fg* show a distinctive mode of chromatin regulation. These clusters are predominantly located within H3K27me3-enriched regions that are largely mutually exclusive with H3K4me3-marked chromatin. Interestingly, even loss of H3K27me3 and associated gene activation at BGCs does not coincide with elevated H3K4me3 levels underscoring the limited understanding of which activating chromatin features are required for BGC expression [18]. This indicates that additional chromatin-based mechanisms are required for BGC derepression, distinguishing their regulation from the canonical histone-mark logic that governs housekeeping genes [7].

Many studies use the host defence compound putrescine as an *in vitro* mimic to induce DON production in *Fg.* In *Fg*, the roles of histone modifications such as H3K27me3 and H3K4me3 have been explored both under putrescine-induced conditions and during actual host infection in two global histone modification analyses [7,19]. Zhao *et al.* [19] conducted Chromatin Immunoprecipitation Sequencing (ChIP-Seq) analysis on *Fg* aerial mycelia at 48 hours post-infection (hpi) scraped from infected wheat spikes, compared to growth on Fusarium minimal media (FMM). Conversely, Shao *et al.* [7] used Cleavage Under Targets and Tagmentation (CUT&Tag) to examine histone modifications *in vitro* under DON-inducing (putrescine) and DON-repressing (NaNO_3_) conditions. CUT&Tag and ChIP-Seq are both antibody-based methods for identification of genomic regions enriched in histone modifications of interest. A more detailed review of these protocols is available here [20,21]. Comparing these datasets reveals how *in planta* infection and chemically defined media shape distinct epigenetic landscapes. However, as this analysis is restricted to a single infection time point and putrescine-based growth, these patterns are likely to vary substantially under different media compositions or infection stages.

In both studies, epigenomic profiling was performed alongside matched transcriptomic analyses under the same experimental conditions. These data demonstrated that enrichment of activating and repressive histone marks correlates with corresponding changes in gene expression. Therefore, although the present analysis focuses on chromatin states, the functional relationship between epigenetic regulation and transcriptional output has been established within each of the two studies analysed here.

**Fig 2:**
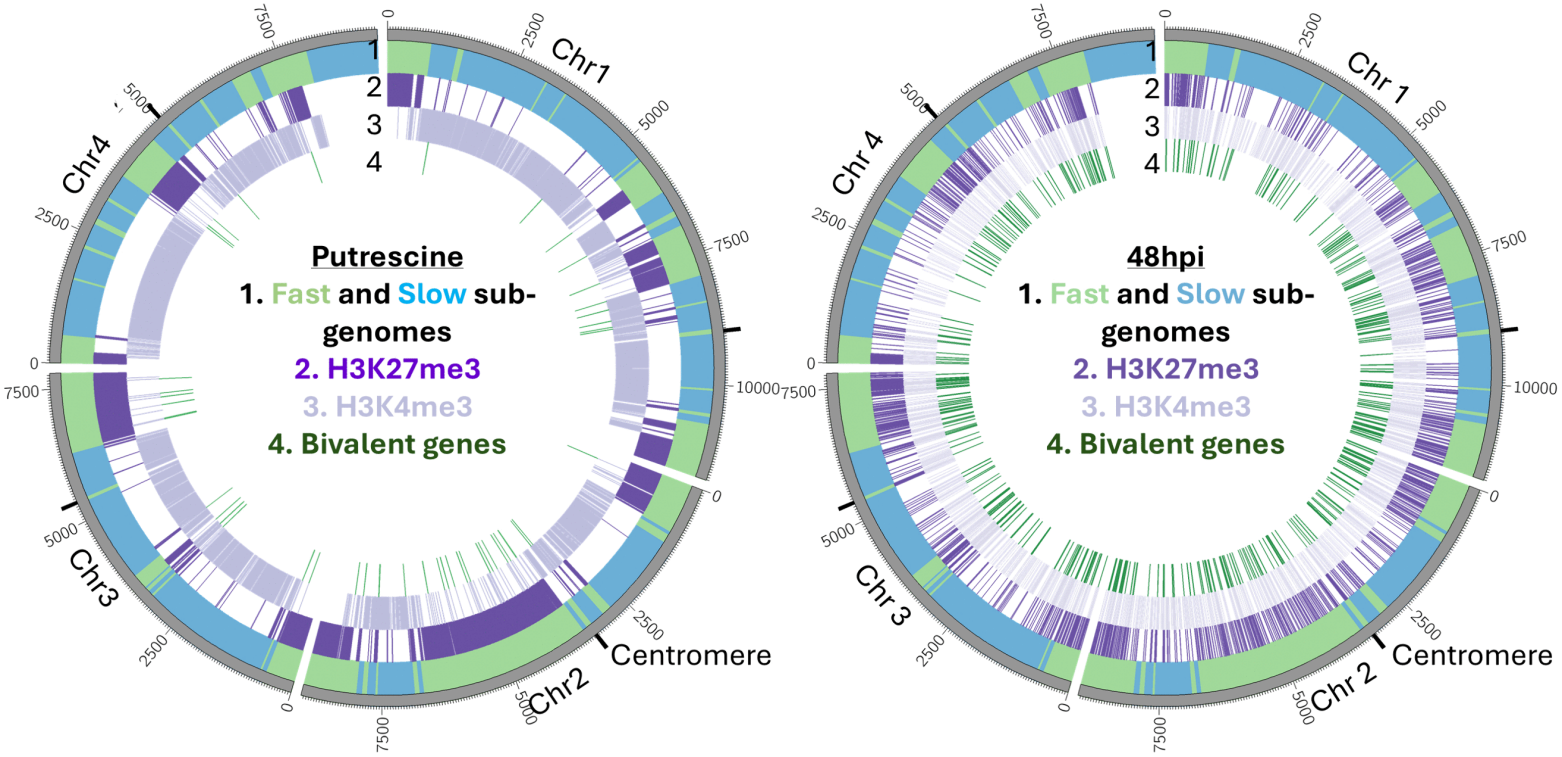
Circos plots showing chromosome scale genome-wide distribution of H3K27me3 and H3K4me3 under two conditions: (Left) *Fg* grown with putrescine (DON-inducing) for 48 hours and (Right) aerial hyphae from infected wheat spikes at 48 hpi. Thick black lines indicate centromeres. Scale is represented in kb. Fast and slow sub-genomes follow Wang *et al*. [29]. Bivalent genes carry both marks. Putrescine: H3K27me3 enriched in fast sub-genome (Z = 5.24, *p* < 0.0001); H3K4me3 enriched in slow sub-genome (Z = 4.09, *p* < 0.0001); opposite associations not significant. Plant associated growth at 48 hpi: Strong enrichment of H3K27me3 in fast sub-genome (Z = 32.90, *p* < 0.0001) and H3K4me3 in slow sub-genome (Z = 3.66, *p* = 0.001); opposite associations were not significant.

To compare both datasets, we have identified methylated regions from the datasets using a PeakCalling pipeline [22]. Briefly, this pipeline retrieves raw ChIP-seq data using the SRA Toolkit [23], performs adapter and quality trimming with Trim Galore [24], aligns reads to the reference genome using Bowtie2 [25], processes and filters the resulting alignments with SAMtools [26] and BEDTools [27], and finally calls peaks and regulatory regions with MACS2 [28]. The genes identified as marked in each condition have been deposited on Zenodo: https://doi.org/10.5281/zenodo.18710745. A genome-wide comparison of the histone-mark distribution revealed major differences between conditions (Fig 2). For the following gene-level comparisons between datasets Chi-squared tests with Bonferroni correction were applied, and associations with the two-speed *Fg* genome [29] were assessed via permutation tests using Z-scores from null distributions.

From this analysis, we observed that only 39% of H3K4me3-marked genes overlapped between putrescine-treated cultures and aerial hyphae at 48 hpi, and just 57% for H3K27me3. Genomic patterns also diverged. Under putrescine, H3K4me3 and H3K27me3 occupied distinct regions with only 48 bivalent genes marked by H3K27me3 and H3K4me3 simultaneously [7], whereas infection samples showed 437 bivalent genes, indicating a more complex regulatory landscape [19].

Notably, the exclusive H3K4me3/H3K27me3 marking under putrescine conditions is consistent with the two-speed genome model for *Fg* [29]. This model proposes that the genome is partitioned into a “fast” sub-genome characterised by high variability and rapid evolution, and a “slow” sub-genome with greater sequence conservation. In this context, H3K27me3 is predominantly associated with the fast sub-genome, which is enriched for secondary metabolite clusters and pathogenicity-related genes [29], while H3K4me3 is more frequently found in the slow sub-genome (Fig 2). At 48 hpi, these associations persisted but were less distinct. Correlation analyses confirmed weak relationships between conditions (H3K4me3: *r* = 0.23; H3K27me3: *r* = 0.27; *p* > 0.05), supporting the conclusion that *in vitro* DON-inducing conditions and host infection produce largely distinct epigenetic landscapes.

These two analyses provide an exemplar for the differences in epigenetic decoration of fungal genomes between distinct infection-mimicking conditions. We demonstrate that histone methylation patterns differ between *Fg* mycelial growth under DON-inducing conditions *in vitro* and aerial mycelia growth collected from infected wheat spikes at 48hpi. Additionally, several factors may contribute to these differences.

Methodological variation could contribute: Zhao et al. [19] used ChIP-seq, while Shao et al. [7] applied CUT&Tag, which in present protocols tends to better detect activating marks such as H3K4me3 [30,31]. However, the contrasting compartmentalisation of H3K4me3 peaks in CUT&Tag versus their more dispersed distribution in ChIP-seq suggests technical bias alone is insufficient.

A more likely explanation is biological. Putrescine-treated cultures experience a simplified stimulus, whereas infection exposes the fungus to diverse plant defences and signals. Consequently, gene sets activated under putrescine differ from those expressed during true infection, reflecting distinct chromatin states and underscoring the role of epigenetic remodelling in rapid transcriptional reprogramming.

Importantly, the analysed mycelial samples are not homogeneous populations. Individual fungal cells are likely to occupy distinct metabolic and developmental states in both putrescine-treated cultures and during infection, meaning that the observed epigenetic profiles represent population-averaged signals rather than uniform chromatin configurations. This cellular heterogeneity should therefore be considered when interpreting differences between experimental conditions and highlights the potential value of single-cell approaches for disentangling stage-specific epigenetic regulation. Although such single-cell epigenomic approaches remain technically challenging in host-associated fungal systems these are highly likely to be pursued in the near future.

While *in vitro* mimics have advanced understanding of DON regulation and chromatin dynamics [7,32–35], these approaches cannot fully capture the complexity of host–pathogen interactions. Similarly, sampling aerial hyphae provides partial insight, but as most fungal hyphal growth occurs within spike tissue, generating datasets from internal wheat spike tissue would greatly improve biological relevance. However, the latter remains technically challenging. ChIP-seq requires large quantities of pure nuclei which are difficult to obtain from mixed samples, whereas CUT&Tag has a low nuclei input requirement and hence offers a promising alternative.

The most stringent comparison between *in vitro* and pathogenic conditions would involve contrasting fungal growth on dead wheat tissue, representing a nondefending but biologically authentic substrate, with growth on and within living wheat cells. This thereby minimises differences in substrate composition while isolating host responses as the primary variable. Notably, a transcriptomic study employing such a design has previously been reported [16] and provides a valuable framework that merits consideration in the context of chromatin-based regulation during infection.

Profiling histone marks across *in host* infection stages could additionally reveal regulatory mechanisms controlling pathogenicity and adaptation, informing disease management strategies. Overall, these findings emphasise that epigenetic landscapes vary dramatically with environment and highlight the need for experimental designs that closely reflect natural infection scenarios.
